# The Role of Cytokinome in the HNSCC Tumor Microenvironment: A Narrative Review and Our Experience

**DOI:** 10.3390/diagnostics12112880

**Published:** 2022-11-21

**Authors:** Nerina Denaro, Cinzia Solinas, Ornella Garrone, Carolina Cauchi, Fiorella Ruatta, Demi Wekking, Andrea Abbona, Matteo Paccagnella, Marco Carlo Merlano, Cristiana Lo Nigro

**Affiliations:** 1 Oncology Department, Fondazione IRCCS Ca’ Granda Ospedale Maggiore Policlinico, 20122 Milan, Italy; 2 Medical Oncology, AOU Cagliari, Policlinico di Monserrato (CA), 09042 Monserrato, Italy; 3 Amsterdam UMC, Location Academic Medical Centre, University of Amsterdam, 1012 WX Amsterdam, The Netherlands; 4 Wellman Center for Photomedicine, Massachusetts General Hospital, Harvard Medical School, Boston, MA 02115, USA; 5 Translational Oncology Fondazione Arco Cuneo, 12100 Cuneo, Italy; 6 Candiolo Cancer Institute, FPO-IRCCS Candiolo (Turin), 10060 Candiolo, Italy; 7 Central Laboratory, Galliera Hospital, 16142 Genoa, Italy

**Keywords:** cytokines, cytokinome, tumor microenvironment, biomarker, head and neck squamous cell carcinoma

## Abstract

Head and neck squamous cell carcinoma (HNSCC) is the sixth most common cancer. In locally advanced (LA) HNSCC, a multidisciplinary approach consisting of surgery followed by chemoradiation (CRT) or definitive CRT is the mainstay of treatment. In recurrent metastatic (R/M), HNSCC immune checkpoint inhibitors (ICIs) with or without chemotherapy represent the new first-line option. However, cancer will recur in about two out of five patients with LA HNSCC. If progression occurs within six months from platin-radiotherapy treatment, anti-programmed cell death-1 (PD-1) may be prescribed. Otherwise, immunotherapy with or without chemotherapy might be considered if PD-L1 is expressed. Despite several improvements in the outcome of patients with R/M HNSCC, overall survival (OS) remains dismal, equaling a median of 14 months. In-depth knowledge of the tumor microenvironment (TME) would be required to change the course of this complex disease. In recent years, many predictive and prognostic biomarkers have been studied in the HNSCC TME, but none of them alone can select the best candidates for response to ICIs or targeted therapy (e.g., Cetuximab). The presence of cytokines indicates an immune response that might occur, among other things, after tumor antigen recognition, viral and bacterial infection, and physic damage. An immune response against HNSCC results in the production of some cytokines that induce a pro-inflammatory response and attract cells, such as neutrophils, macrophages, and T cell effectors, to enhance the innate and adaptive anti-tumor response. We revised the role of a group of cytokines as biomarkers for treatment response in HNSCC.

## 1. Introduction

Head and neck squamous cell carcinoma (HNSCC) is a heterogeneous disease arising from the oral mucosa of the first aerodigestive tract in which tobacco, alcohol, and the human papillomavirus (HPV) infection are well-known causative factors [1]. In recent decades, the treatment of HNSCC has evolved thanks to technical advances in radiotherapy techniques and multidisciplinary approaches. However, the standard treatment for LA HNSCC has been unchanged in the last twenty years. For the recurrent/metastatic (R/M) HNSCC, the standard of care has significantly changed in the last few years following the introduction of immune checkpoint inhibitors (ICIs), given alone or in association with chemotherapy. Nevertheless, modest improvements are still seen [2].

The standard first-line therapy includes immunotherapy alone (Pembrolizumab) or in combination with Platin and 5-Fluorouracil and achieves a median overall survival (OS) of about 14 months, with a response rate (RR) ranging from 20 to 43% and a duration of response of 7–20 months in patients with PDL-1 combined positive score (CPS) of 1–20%.

The link between an efficient anti-tumor inflammatory response and the disease outcome has been established in solid tumors through experimental evidence demonstrating an influential role of pro-inflammatory cytokines on tumor growth and progression. Over recent years, many inflammatory cytokines (IL-6, IL-8, VEGF, TNF, etc.) have been identified as prognostic predictors in colon, breast, and lung cancers [3]. Therefore, cytokines represent molecular actors of tumor growth and progression.

The tumor microenvironment (TME) is a dynamic context in which stromal, immune, and tumor cells interact and it is critical for effective therapy with ICIs. HNSCC evades growth inhibitory effects of anti-tumor cytokines through a complex network of proliferation and regulatory factors. As well as other solid tumors, HNSCC secretes cytokines not only to inhibit the TME but also to recruit immune-suppressive cells that secrete other pro-tumoral cytokines [4].

The term “cytokinome” is used to indicate the evaluation of cytokine pattern by using an “omics” approach [5]. Cytokinome detects the whole set of cytokines in a biological fluid (in plasma or whole blood) at a certain moment. Its most interesting advantage is that cytokines could be considered as molecular biomarkers of the ongoing immune response and that they could interact with proliferation pathways such as angiogenesis, BRAF, MEK, and their downstream genes. In spite of their potential, the application is limited by the cost of techniques [5].

Established devices for cytokine detection include ELISA, fluorescent-based immunoassay, immune-PCR, non-antibody spectrometry, electrochemical aptamers, and affinity mass spectrometry. New emerging assays are under investigation.

The advantage of utilizing the cytokinome approach is allowing the serial determination of multiple cytokines to compare the physiological dynamics of the whole set. Indeed, classical single cytokine assays were not representative because the values of a single cytokine are likely not capable of capturing small changes in the microenvironment in both quantity and time. Identifying a restricted group of cytokines might be relevant for personalized therapies to manipulate the ongoing immune response with immunotherapy.

Recently, the role of Cytokines/Cytokinome as a predictive biomarker for cancer therapeutics in solid tumors has been identified. Huntington et al. [6] analyzed the cytokine response when colorectal tumor cell lines were treated with various anti-tumoral small molecules. MEK-inhibitors downregulated VEGF, CXCL9/MIG, and IL-8/CXCL8 and upregulated CXCL14/BRAK, prolactin, and CCL-5/RANTES. BRAF-inhibitors down-regulated VEGF and IL-8/CXCL8, while increasing soluble TRAIL-R2. Treatment with PARP-inhibitors decreased CXCL9/MIG, IL-8/CXCL8, CCL-3/MIP-1 alpha, VEGF, and CXCL14/BRAK, while the treatment increased soluble TRAIL-R2 and prolactin. Moreover, Huntington et al. [6] confirmed heterogeneity in cytokine, chemokine, and growth factor responses across cell lines and drug treatments in colorectal cancer. Considering Huntington’s work is in vitro it is possible, however, to speculate that since there is no clear division among biomarkers related to immunosuppression or unfavorable prognosis, and those correlated with immunostimulation, a favorable prognosis could be determined.

In order to optimize treatment efficacy and minimize toxicities from these treatments, there is an urgent need to select ideal candidates, based on individual biomarkers for anti-programmed cell death-1 (PD-1), ICIs alone or in combination, and to verify if the sequence of chemotherapy and ICIs might be conceivable in HNSCC patients.

HNSCC has been separated into high– and low–risk stratifications using an immune-related gene signature (IRGS) from two transcriptional datasets: The Cancer Genome Atlas (TCGA) and Gene Expression Omnibus (GEO). IRGS considers all cytokines active in TME. The IFN-α response, the IFN-γ response, IL-2/STAT5 signaling, and IL-6/JAK/STAT3 signaling were all negatively correlated with outcome [7].

Findings on cytokine effects and outcomes have been reported since 2000. For instance, for patients in intensive care units, cytokines IL-8, IL-6, and IL-10 were related to mortality, and patients with sepsis and low cytokine levels had a dismal prognosis [8].

Since cytokinome plays a significant role in identifying and characterizing individual prognostic and predictive biomarkers, we decided to review the recent literature on this topic including preliminary results from our group presented in recent outstanding international meetings. At Translational Oncology Laboratory Fondazione Arco Cuneo, we analyzed R/M HNSCC patient samples to test our hypothesis that identifying groups of cytokines could define different risk populations. In this paper, we review published papers investigating the role of cytokinome in HNSCC.

Cytokinome is known as a biomarker of TME. In this paper we suggest its role as a prognostic predictor in HNSCC.

## 2. Materials and Methods

### 2.1. Literature Review

The MEDLINE database was searched for studies published from January 2000 to July 2022 containing the terms “tumor microenvironment, cytokine levels, cytokinome, cytokine profile, head and neck cancer.”

The literature search was limited to articles written in English about detecting cytokine levels in patients diagnosed with HNSCC and other solid tumors. Potentially relevant abstracts presented at annual meetings of the American Society of Clinical Oncology (ASCO), American Association of Cancer Research (AACR), and the European Society of Medical Oncology (ESMO) were also examined. The study selection criteria included the following: (a) observational and prospective studies about the assessment of the levels of cytokines in HNSCC; (b) studies on cytokines in HNSCC; (c) retrospective and uncontrolled studies; (d) systematic reviews and meta-analyses. Furthermore, the electronic search results were supplemented by a manual examination of reference lists from selected articles.

### 2.2. Our Experience in the Analysis of Cytokinome in R/M HNSCC

We collected peripheral blood samples for cytokine analysis in patients treated with immunotherapy (nivolumab, Opdivo, Bristol-Meyer) at baseline. We selected 18 cytokines: IL-2, IL-4, IL-5, IL-6, IL-8, IL-10, IL-12, IL-13, IL-15, IL-21, CCL-2, CCL-4, CXCL-10, CCL-22, TGF-β, TNF-α, VEGF and IFN-γ. Concentrations of all cytokines except for IL-21 were measured using the Ella Simple Plex system (ProteinSimple™, San Jose, CA, USA) according to the manufacturer’s instructions. IL-21 was assessed using ELISA method (R&D System Minneapolis, MN, USA).

Differences in the median cytokine values or cell populations were analyzed using nonparametric Mann-Whitney U test and Wilcoxon signed-rank test for paired samples.

The patient population was divided into two groups depending on the value of cytokines at T0 using cut-off points calculated by receiving operating curve (ROC) analysis. Principal component analysis (PCA) was performed to group patients with good or poor PFS and OS. In all statistical tests, *p* < 0.05 was considered to indicate significance.

## 3. Results

**Figure 1:**
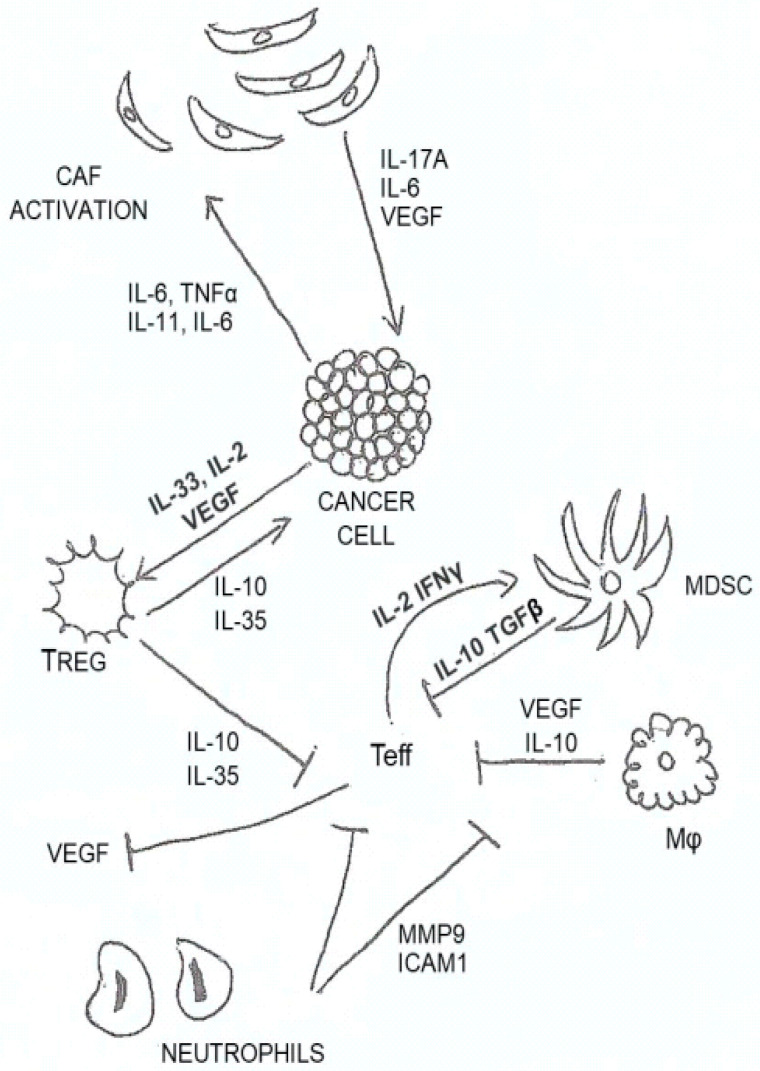
Most relevant cytokines in HNSCC. Th1 produces IL-2 and IFN-γ and recruits macrophages; Th2 produces IL-4, IL-5, and IL-13 and recruits eosinophils and basophils; Th17 produces IL-17, IL-21, and IL-22 and recruits neutrophils; cytotoxic T cells produce IFN-γ and perforins and recruits NK cells; macrophages produce TNF, IFNa/b, IL-6, and IL-1b and recruits Tregs and MDSCs; neutrophils produce MMPs, ROS, peroxidase, IL-1, IFNg and TNF and recruits Tregs and MDSCs.

In Figure 1, we illustrate and summarize the most important cytokines involved in HNSCC and their role in the TME.

### 3.1. Literature Revision

A recent review by Kartikasari et al. [9] demonstrated the predictive role of some cytokines in cancer detection and outcome; in particular, NFkB through IL-6, TNF, IL-8, IL-17, IFN-γ, and CCL-5 correlate to cancer progression [10]; IL-6 via STAT3, has been shown to promote tumor cell proliferation in many cancers but also chemotherapy resistance (HNSCC, colon, and prostate cancers). Furthermore, Kartikasari et al. [9] reported IL-6, IL-1β, and TNF-α to induce epithelial-mesenchymal transition; IL-6, IL-13, IL-4, and IL-10 to cause resistance to therapy. High levels of IL-6 correlate to cancer metastasis, while high IL-6 and IL-17 predict cancer recurrence after radical treatments. Several cytokines (IL-4, IL-10, IL-13) have been shown to confer drug resistance; their mechanism is not fully understood, but an anti-apoptotic effect has been suggested. Lastly, cytokine clusters have been associated with circulating cancer-specific proteins such as cancer antigen (CA) 19–9, carcinoembryonic antigen (CEA), and CA-724 to detect gastrointestinal cancers and ovarian in early stages [11,12,13].

### 3.2. Our Experience in the Analysis of Cytokinome as an Effort to Find Prognostic Biomarkers

In the Laboratory of Translational Oncology at S. Croce & Carle Teaching Hospital in Cuneo (Italy), we developed an ELISA method for detection purposes. We determined levels of pro-inflammatory cytokines in cancer patients at the following time points: pre-treatment, during therapy, and at the end of treatment. Our previous work with breast cancer patients demonstrated that high levels of TGF-β, TNF-α, VEGF, IL-6, IL-8, and IL-10 correlated with poor prognosis, while IL-21 and CCL-2 are related to a better outcome. In particular, patients with early clinical progression during eribulin therapy had an increase of TGF-β, while responders had low levels of TGF-β and IL-8 and high levels of IL-21 [14].

**Figure 2:**
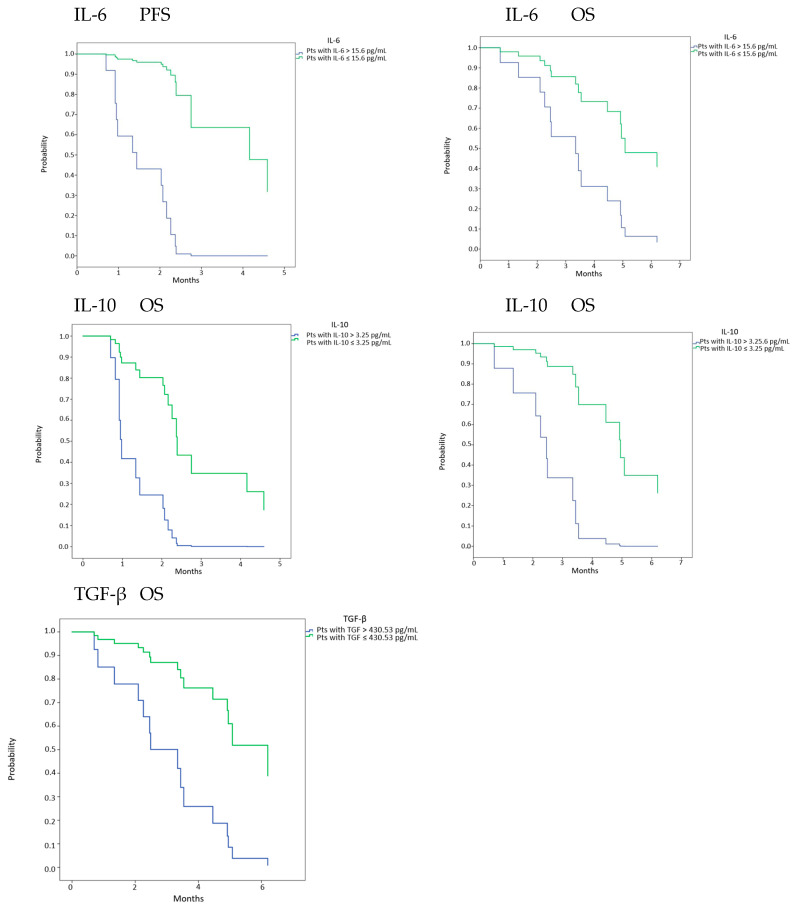
PFS and OS in pts with baseline cytokines levels lower or higher than cut-off point. Cut-off points for each cytokine were calculated using ROC analysis. OS and PFS were expressed in months.

**Figure 3:**
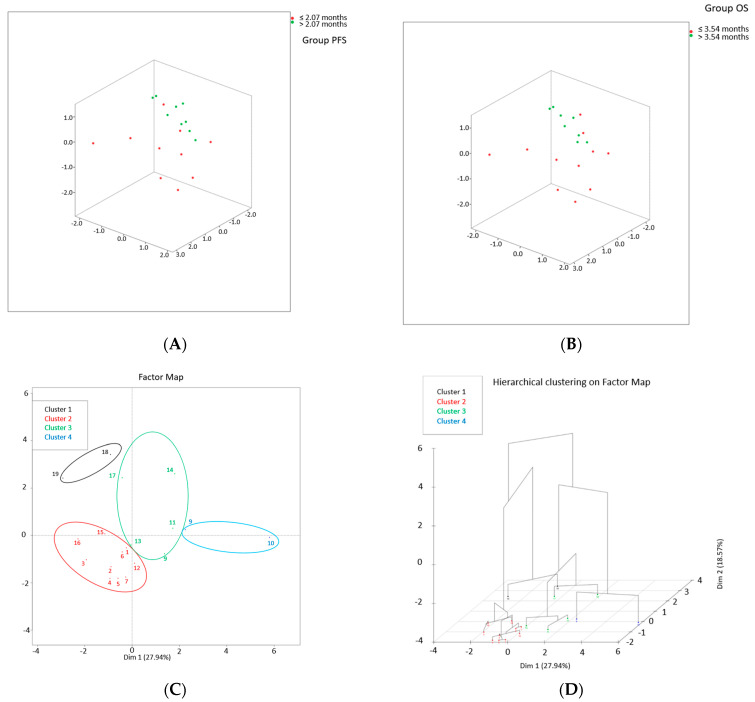
Cluster analysis. (**A**) Tridimensional PCA plot [pts with PFS <= median (red); pts with PFS > median (green)]. (**B**) Tridimensional PCA plot [pts with OS <= median (red); pts with OS > median (green)]. (**C**) Factor map of K-means cluster and (**D**) HCPC 3D dendrogram showing the 4 clusters of pts (cluster 1: black; cluster 2: red; cluster 3: green; cluster 4: blue).

These data were further confirmed in HNSCC (Figure 2 and Figure 3). In the Nivactor trials, a translational study analyzed cytokines as biomarkers. These results will be published soon. A cluster including IL-6, IL-8, IL-10, TGF-β, CD3 + CD8 + LAG3 +, and CD33 + CD11 + HLA-DR high CD14 + was able to discriminate those at good versus bad prognosis (Merlano M unpublished).

Moreover, in the advanced setting of pluri-treated HNSCC patients, we demonstrated among a panel of 17 cytokines, that high IL-2, low IL-8, and CCL-2 correlated with OS, and that increases in IL-10, IL-8, and CCL4 correlated with poor outcomes (14). Data from 19 SCCHN pts were analyzed; medians of PFS and OS were 2.07 months and 3.54 months, respectively.

Using ROC analysis, that we could identify cut-off values at basaline which correlated with PFS and OS. IL-6 and IL-10 (15.6 pg/mL, 3.25 pg/mL), and TGF-β only correlated with OS (420.53 pg/mL) (data non shown). Then, performing Cox analysis, it was found that patients with IL-6 or IL-10 lower than cut-offs had significant better PFS (HR 0.008 95% C.I. 0.000–0.981, *p* = 0.049 and HR 0.157 95% C.I. 0.045–0.543, *p* = 0.003). Moreover patients with IL-6, IL-10 or TGF-β lower than cut-offs had significant better OS (HR 0.285 95% C.I. 0.092–0.882, *p* = 0.029; HR 0.101 95% C.I. 0.020–0.510, *p* = 0.006; HR 0.201 95% C.I. 0.053–0.755, *p* = 0.02) (Figure 2).

Moreover, using hierarchical cluster on principal components (HCPC), with 3 PCs, we identified 4 clusters of patients using as parameters VEGF, CCL-4, CXCL-10, TGF-β, IL-5, IL-6, IL-10, IL-12, TNF-α, CD8+ (Naïve, CM, TEMRA, LAG3+). Cluster 2 has the best PFS and OS compared to the remaining clusters (HR 0.107 95% C.I. 0.027–0.416, *p* = 0.001 and HR 0.045 95% C.I. 0.005–0.380, *p* = 0.004, respectively) (Figure 3) [15].

In Table 1, we reported the action of a group of cytokines correlated to treatment response in solid tumors and HNSCC.

## 4. Discussion

Cytokines are regulatory proteins involved in both host defense and normal/abnormal homeostatic mechanisms. They have two main peculiarities: redundancy (same effects exerted by different molecules) and pleiotropy (different effects exerted by the same protein) [36]. Cytokines are secreted in response to a stimulus from immune cells or tumoral cells. Under certain conditions, cytokines can generate a forward feedback loop to stimulate self-proliferation and recruit immune cells inducing a vicious circle (immune cells produce other cytokines than recruit immune cells and so on).

Therefore, cytokines are key players in response to the tumor microenvironment as they may exert pro- and anti-tumoral effects. Some suppress tumor formation by controlling infection, inflammation, and immunity, while others promote growth, increase resistance to apoptosis, and foster dissemination. The pro- and anti-tumoral effects might co-exist in different phases of the disease.

Traditionally cytokine classification involves several characteristics (such as targets, cells of origin, and spectrum of activity). Pries et al. [17] classified HNSCC-relevant cytokines based on their action: angiogenesis (FGF, IL-8, HGF, VEGF, PDGF) and immune suppression (PGE2, TGF-β, IL-10, IL-4, GM-CSF). Several other classifications have been proposed during the last two decades as well. However, none of these classifications fully reflect the factors in the HNSCC TME. For our study, based on the literature review, we considered their classification as pro-tumoral (TNF-α, TNF-β, VEGF, IL-lα, IL-6, IL-1/3,TGFα) or anti-tumoral (IL-6, TNF-α, IL-1ß, IL-18, IL-12, IL-2, IFN-α, IFN-β, and IL-12) [37].

The double role of various cytokines in the HNSCC TME is typical, and the most relevant cytokines are described in this section. IFN-γ has an important anti-tumoral effect, but it also causes an increase in PD-L1 and stimulates cancer cells [38]. IL-2, on the one hand, increases T effectors and NK cells, and on the other, increases T-regs [39]. The role of IL-17 is controversial. On the one hand, it exerts anti-tumor activity in melanoma and ovarian cancers; on the other hand, it induces immunosuppression in breast cancer and HNSCC. High levels of IL-17 with high levels of T cells correlate with a good prognosis, but low tumor-infiltrating lymphocytes with high levels of IL-17 are associated with bad outcomes [40]. IL-1α stimulates immunosuppression and increases the production of CAFS and chemokines such as CCL7, CXCL-1, and IL-8. Moreover, high levels of IL-1α are associated with hypoxia [41]. IL-1β correlates with epithelial-mesenchymal transition, leading to downregulation of E-cadherin and increased levels of T-reg cells. Notably, it has been hypothesized that upregulation of IL-1 is a predictive factor of response to Cetuximab [42].

More than ten years ago, Scheller J et al. demonstrated that the dual response to IL-6 secretion depends on trans versus classic signaling. As for IL-17 and TGF-β, the multifunctional effect depends on the involved cells. For example, TGF-β produces an immunosuppressive effect in NK cells but not in Th17 cells [43].

Among pro-inflammatory cytokines, high IL-8 is associated with bulky nodal involvement and plays an important role in cancer invasion, angiogenesis, and metastasis. It correlates with short PFS and OS [44].

The advantage of evaluating and identifying cytokines as biomarkers is the non-invasive approach; however, several assay platforms that differ in the number of targets, experiment time, and sensitivity are available for the cytokine analysis.

In HNSCC TME, stromal, immune, tumor cells, and extracellular matrix complex interact in a complicated network. The proportion of immune T and non-T cells changes over time towards tumor progression. In the most advanced diseases, non-T immune cells prevail over T cells. These connections/exchanges condition many different pathogenic cytokine levels, which can generate an immunosuppressive or exhausted microenvironment [45]: the level of cytokines is a thermometer in the TME.

The HNSCC TME differs between HPV-positive and negative tumors. In HPV-positive tumors, the TME is enriched in CD3^+^ T cells (including both CD4^+^ T helper and CD8^+^ cytotoxic T cells), NK cells, MDSCs and DCs. Further, there is a higher secretion of IL-10, CCL2, IL-6, TGF-β, TNF-α, and EGF compared to HPV-negative TME [46].

Pro-tumoral macrophages produce IL-6 and IL-10 that stimulate MDSCs and TGF-β and induce epithelial-mesenchymal transition, with an immunosuppressive effect. Moreover, IL-6, IL-17A, IL-22, growth factors such as EGF, HGF, VEGF, chemokines such as C-X-C motif chemokine ligands (CXCLs), CXCL-1, CXCL-8, CXCL-12 (SDF-1α) and CXCL-14, and C-C motif chemokine ligands (CCLs), CCL-2, CCL-5, and CCL-7 are involved in the recruitment of cancer-associated fibroblasts (CAFs). IL-33 is a critical mediator in CAF-induced invasiveness [47].

CAFs are associated with tumor progression and metastasis and induce chemoresistance through angiogenesis and extracellular matrix modeling [48]. Inflammation, chemotherapy, and ICI cause alteration in the microenvironment and their efficacy relies on the composition of CAFs.

Unfortunately, a pro-inflammatory cytokine panel is an index of the presence of chronic inflammatory disease, including neoplastic disease, but not of a specific one. Moreover, we have not yet defined a peculiar pattern of cytokines particular to HNSCC (prognosis and predictive markers), possibly because both disease heterogeneity and cytokine levels change over time.

In the last few years, several scientists have clustered specific cytokines to classify the chronic inflammatory disorders that share similar pathogenic pathways in the context of resident tissue cell lineages, such as rheumatoid arthritis [49].

Al-Yahya S et al. [50] demonstrated cytokine modulation of type I and II IFN responses. IFNs and cytokines were able to augment IFN-α or IFN-γ responses by their ability to directly activate stimulating responsive elements (ISRE) or gamma-interferon-activation sites GAS responses. Nonetheless, the functional redundancy of the pathways in which the cytokines are involved, also due to the structural pleiotropy of many cytokines, made it difficult to specifically target the key cytokines, even with specific antibodies.

Wong et al. [51] performed a multiplatform integrative analysis of 261 cytokines and cytokine receptor alterations across 19 human cancer types. Cytokine levels correlate with advanced stage and symptoms. Pro-inflammatory cytokines are associated with fatigue, depression, and cognitive impairment and may affect the quality of life before, during, and after treatment.

In more advanced cancer, pro-inflammatory cytokines are additionally associated with anorexia and cachexia, pain, treatment toxicity, and resistance to treatment [3]. In the geriatric setting, a signature of physical frailty and sarcopenia has been validated by an Italian group. Cancer patients are elderly and frail; this signature might reflect the cytokinome of the patients. Moreover, among the elderly higher levels of p-selectin, C-reactive protein (CRP), and interferon γ-induced protein 10 correlated with sarcopenia and worse performance status [52].

## 5. Conclusions

In conclusion, our literature review on cytokines and cytokinome suggest the potential role of these factors as predictive and prognostic biomarkers.

Although time-dependent, the cytokinome approach by multiplexing measurements might be advantageous in identifying cytokine clusters that might be specifically targeted with, for example, antibodies or other compounds. The actuality of this approach is demonstrated by the increasing number of publications on this topic. Cytokines mirror the TME; emerging biomarkers are therefore based on these components in the TME and include cytokine sets, combined cytokines and their soluble receptors, combined cytokines, and cancer-derived proteins.

In order to optimize the benefits and minimize toxicities from cancer therapies, we need valid predictive and prognostic biomarkers to select patients to receive anti-PD-1 alone or in combination, and to identify patients as candidates for de–escalation or intensification of cancer treatment.

Using a cluster of cytokines to individualize treatments and prognosis in HNSCC is a novel strategy that requires further investigation.

## Figures and Tables

**Table 1:** Cytokines correlated to treatment response in solid tumors and HNSCC.

Reference	Role/Action	Cytokine/Chemokine
[16,17]	TNF-β is secreted by T and B lymphocytes. It shares approximately 30% structural homology with TNF-α. It plays an important role in host defense against infection and the growth of some tumors.	TNF-β
[16]	As TNF-β, TNF-α exerts a wide variety of effects on diverse cell types through gene modulation of growth factors, cytokines, and transcription factors. In particular, like IL-1, TNF-α is a macrophage product and has the property to induce bone resorption, procoagulant activity, fibroblast growth, and expression of adhesion molecules on endothelial cells. TNF-α induces depletion of IFN-γ. Recently, TNF patterns were used to score HNSCC. High expression of TNF family members correlates with better responses. A favorable pattern is associated with p53 negative, not EGFR amplification, and HPV positive tumors. Additionally, TNF-α is correlated with CD4 and CD8 cell infiltrations.	TNF-α
[18]	VEGF is the key mediator of angiogenesis in cancer, in which it is upregulated by oncogene expression, a variety of growth factors, and hypoxia. It induces epithelial to mesenchymal transition.	VEGF
[19]	IL-6 is produced by macrophages and monocytes, fibroblasts, activated T lymphocytes, and endothelial cells (in response to IL-1 and TNF-α. IL-6 causes hepatocytes to synthesize plasma acute phase proteins and is a growth factor for malignant plasma cells and hematopoietic stem cells.	IL-6
[20,21]	IL-8 is secreted by monocytes, fibroblasts, epithelial cells, astrocytes, keratinocytes, synovial cells, and various tumor cells. It has chemotactic receptor agonist property and causes neutrophils’ shape changes, chemotaxis, and exocytosis. IL-8 increases angiogenesis and correlates with high macrophage infiltrates.	IL-8
[22,23]	IL-10 is secreted by activated T cells. It can inhibit both T cells and NK cells and macrophage function (reducing the secretion of cytokines from Th1 T-cell clones and macrophage functions, including microbicidal properties). It stimulates B cells.	IL-10
[24]	IL-21 is secreted by activated T cells and NK cells; it regulates a wide range of immune cells, including T and B cells, NK cells, DCs, and macrophages, as well as non-immune cells, including epithelial cells and keratinocytes. It activates the Janus kinase (JAK1/3)-signal transducer and activator of transcription (STAT) signaling pathway.	IL-21
[25]	CCL-2 recruits monocytes, memory T cells, and dendritic cells to the sites of inflammation produced by either tissue injury or infection. CCL-2 is implicated in the pathogenesis of several diseases characterized by monocytic infiltrates. It correlates to macrophage accumulation in cancer.	CCL-2
[26,27]	TGF-β is able to induce both pro- and anti-tumoral effects. It affects proliferation and differentiation in a wide variety of cell types. It regulates extracellular matrix proteins and cell adhesion. It affects mesenchymal differentiation and is a potent chemotactic agent for various cell types, including monocytes and fibroblasts. It suppresses the activity of B and T lymphocytes, macrophages, and NK cells, regulating cytokine production by different cell types.	TGF-β
[28,29]	IL-2 is secreted by activated T cells, NK cells, and dendritic cells. It is known for its pleiotropic effect. It can promote T-cell and NK cell cytotoxicity activity and modulates T-cell differentiation programs in response to antigens. It has been used for the therapy of renal cell carcinoma and melanoma. In vitro IL-2 induces regression in a few HNSCC patients.	IL-2
[30]	It is mainly secreted by activated T cells, NK cells, eosinophils, and basophils. Its activity is correlated to IL-13. It regulates antibody production, hematopoiesis, and inflammation and is also involved in developing effector T-cell responses.	IL-4
[31]	IL-5 is produced mainly by lymphocytes, eosinophils monocytes, and macrophages. IL-5 stimulates antibody, eosinophil differentiation and proliferation and tumor cell migration and activation through STAT5 signaling.	IL-5
[32]	IL-12 is a potent, pro-inflammatory cytokine. It has anti-tumor activity, and it counteracts IL-23 effect. It increases activation and cytotoxic capacities of T and NK cells and inhibits pro-tumoral macrophages and myeloid–derived suppressor cells.	IL-12
[33]	IL-13 inhibits macrophage production of TNF, IL-1β, and pro-inflammatory chemokines, but can upregulate the synthesis of IL-12 by DCs and macrophages. IL-13 impairs antibody-dependent cytotoxicity. IL-13 stimulates B cell activation. IL-13 also promotes isotype switching to IgE and IgG1 and matrix expression (VCAM-1).	IL-13
[34]	IL-15 is an inflammatory cytokine. It is secreted primarily by monocytes and macrophages. It stimulates TNF-α, IL-1β, inflammatory chemokines, T and B cells, and NK cells. It is regulated post-transcriptionally at the levels of translation and intracellular trafficking.	IL-15
[35]	IL-1 stimulates CAFs, which produce CXCL1, CXCL8, and MMP-1. IL-1β increases neutrophils to the TME, and neutrophil accumulation has been related to poor outcomes in HNSCC. IL-1^2^ stimulates VEGF and recruits T regulatory cells.	IL-1β

CAF, Cancer-Associated Fibroblast; CCL—Chemotaxis towards CC chemokine Ligand; CXCL, chemokine (C-X-C) Ligand; DC, Dendritic Cell; HNSCC, Head and Neck Squamous Cell Carcinoma; HPV, Human Papilloma Virus; IFN, Interferon; IL, Interleukin; JAK-STAT, Janus Kinase-Signal Transducer and Activator of Transcription; MMP, Matrix Metalloproteinase; NK, Natural Killer; TGF, Tumor Growth Factor; TME, Tumor Microenvironment; TNF, Tumor Necrosis Factor; VCAM—Vascular Cell Adhesion Molecule; VEGF, Vascular Endothelial Growth Factor.

## Data Availability

Details regarding the study design, statistical Analysis supporting results and informed consent are reported in the Study approved by S. Croce & Carle Ethical Committee at February 2017 (2017-000562-30).
